# METTL14 Induced N^6^-Methyladenosine Modification of FOXP4 mRNA in HBV-HCC

**DOI:** 10.7150/jca.101385

**Published:** 2024-10-14

**Authors:** Tian-Tian Wang, Yi-Mei Ji, Qian Zhang, Bo Liang, Ting-ting Fan, Xin Ye

**Affiliations:** 1 Department of Endoscopy, Eastern Hepatobiliary Surgery Hospital, Second Military Medical University, Shanghai, 200438, China.; 2 Department of General Surgery, The Second Affiliated Hospital of Nanchang University, Nanchang University School of Medicine, Nanchang, Jiangxi Province, China.

**Keywords:** epitranscriptomic modification, HBV, Foxp4, METTL14, HCC

## Abstract

Chronic hepatitis B virus infections are a significant cause of liver cirrhosis and cancer. Our research reveals that HBV infection leads to a marked increase in m6A modification of Foxp4 mRNA, resulting in enhanced stability of the mRNA and a subsequent increase in Foxp4 mRNA levels. Analysis of biopsy samples from chronic HBV patients demonstrated consistent upregulation of m6A-modified Foxp4 mRNA levels alongside increased Foxp4 mRNA levels. Functionally, Foxp4 was found to promote proliferation, migration, and invasion of hepatocellular carcinoma (HCC) cells in laboratory settings. Additionally, HBV gene expression was shown to activate the PI3K/AKT pathway by modulating Foxp4 mRNA stability in HCC cells. This study provides valuable insights into the underlying mechanisms of HBV infection and its potential implications for cancer development.

## Introduction

Hepatitis B virus (HBV) infection is a serious concern as it can progress to chronic hepatitis and increase the risk of developing HCC 1-3. Research suggests that HBV can manipulate the host's gene expression to facilitate viral replication and ensure its survival 4. This manipulation ultimately contributes to the progression of chronic hepatitis B to liver cirrhosis and potentially HCC 5. However, the specific mechanisms or triggers that lead to HBV-related HCC are not yet fully understood.

Viruses have the ability to manipulate host gene expression profiles, facilitating their own replication and establishment within the host 6-9. A common epigenetic alteration employed by viruses is the modification of RNA, specifically the N6-methyladenosine (m6A) modification of mRNAs 10-13. Foxp4 plays a critical role in tumorigenesis and cancer progression 14-17. In this study, we delve into the HBV's interaction with the host gene Foxp4, a key player in tumorigenesis and cancer progression. We reveal that HBV specifically targets Foxp4 mRNA for m6A modification, resulting in a stabilization of Foxp4 mRNA levels. The identified mechanism suggests a novel avenue for HBV-associated hepatocellular carcinoma (HCC) development.

## Materials and Methods

### Cell culture and transfection

Hepatocellular carcinoma (HCC) cells from ATCC were cultured as outlined previously. Primary human hepatocytes were grown following the manufacturer's instructions. Lipofectamine RNAiMAX was used to transfect shRNAs as per the protocol 18. The OE-NC, OE-Foxp4, sh-NC, sh-Foxp4, sh-METTL3, sh-METTL14, sh-FTO, sh-KIAA1429, sh-ALKBH5, and HBV 1.3-mer, FLAG-YTHDF1, 2, and 3 plasmids were acquired from Shanghai Yuan Zhi Biotechnology Co., LTD. The effects of stable knockdown and overexpression effect are shown in Supplementary Figure 1. shRNA sequences used for genes knockdown was listed in Supplementary table 1.

### qRT-PCR

RNA isolation was performed with Trizol reagent, and qPCR was conducted using SYBR Green supermix following the manufacturer's instructions.

### Cell proliferation assays

We employed the YF 555 Click-iT EdU kit (C6016L, US Everbright® Inc., China) to conduct the EdU assay following the manufacturer's instructions.

### Cell migration & invasion assays

To assess cell migration and invasion was performed following the manufacturer's instructions. Finally, the migrated/invaded cells were counted using an inverted microscope.

### Statistical analysis

All presented results were replicated in three independent experiments. Statistical significance was determined using a one-tailed unpaired Student's t-test.

## Results

### HBV modulates m6A modification on Foxp4

**Figure 1:**
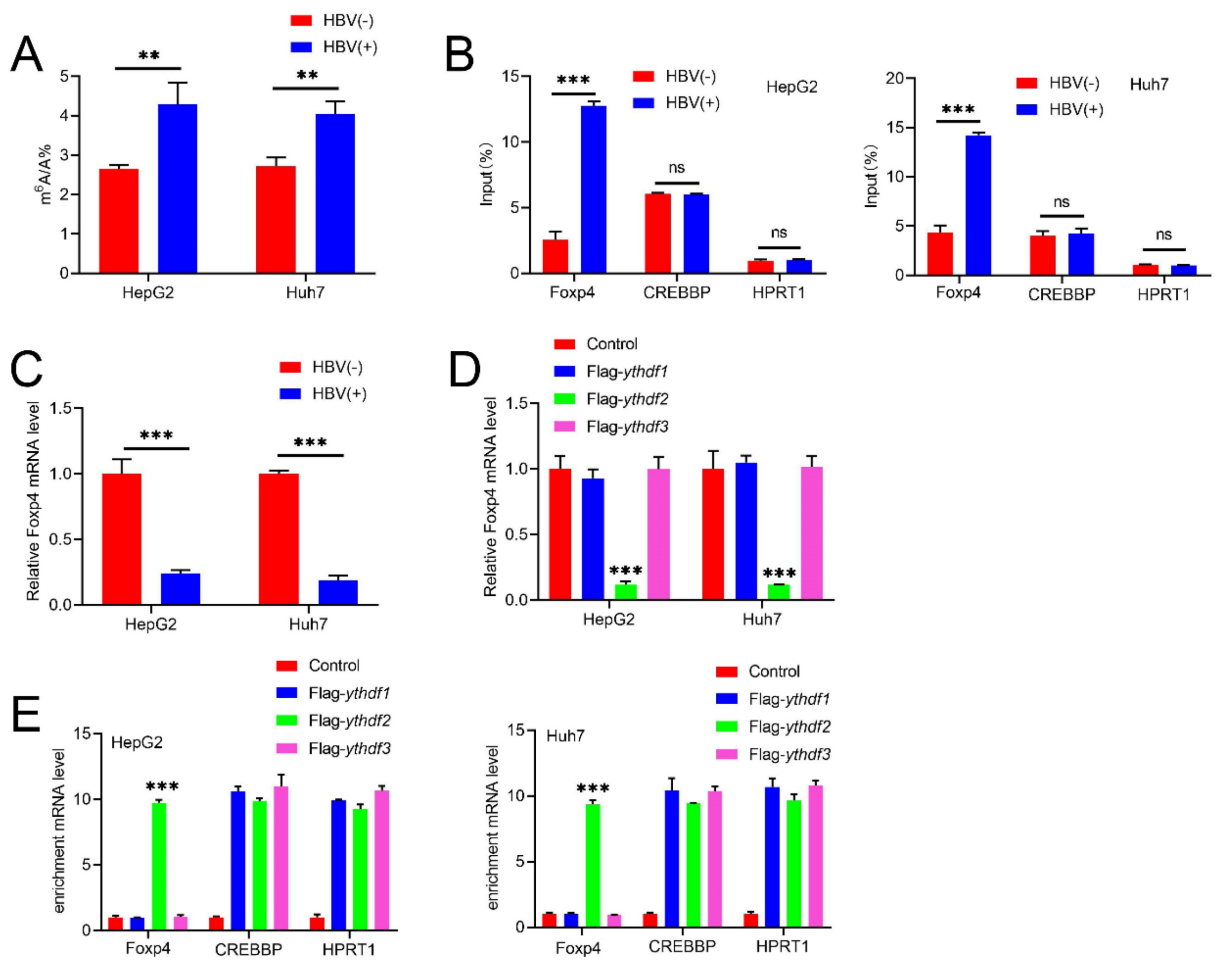
** HBV modulates m6A modification on Foxp4.** (A) m6A level in indicated HCC cells. (B)MeRIP-PCR was performed on m6A modified HBV transcripts and Foxp4 mRNA. (C)The mRNA level of Foxp4 in HCC cells was determined using qRT-PCR. (D) Enriched Foxp4, CREBBP, and HPRT1 mRNA was quantified by qRT-PCR. (E) HCC cells were transfected with FLAG-YTHDF2 and Foxp4 mRNA quantified. Ns P > 0.05; **P <0.01; ***P < 0.001.

LC-MS/MS assay revealed a significant increase in m6A abundance in HBV-infected HCC cell lines (Figure.1A). To further investigate the mechanism, we conducted MeRIP assays, which showed that HBV transfection elevates m6A modification levels of Foxp4 mRNA. To validate our findings, we used CREBBP (a cellular RNA known to be modified by m6A) as a positive control and HPRT1 (a cellular RNA that lacks m6A modification) as a negative control 19. The results indicated that HBV-induced m6A modification of Foxp4 mRNA positively regulates its expression levels (Figure 1C). The stability and translation of m6A-modified RNAs are governed by "reader proteins" such as YTHDFs 20. FLAG-YTHDF1 immunoprecipitated samples demonstrated an enrichment of Foxp4 mRNAs compared to control samples and those immunoprecipitated with FLAG-YTHDF2 or FLAG-YTHDF3. CREBBP and HPRT1 RNAs served as positive and negative controls, respectively (Figure 1D). Transient expression of YTHDF1 in HCC cells resulted in elevated Foxp4 mRNA levels (Figure 1E). Collectively, these results establish that Foxp4 mRNA undergoes m6A modification, which is induced by HBV infection.

### HBV enhances FOXP4 mRNA stability via modulation of m6A modification

**Figure 2:**
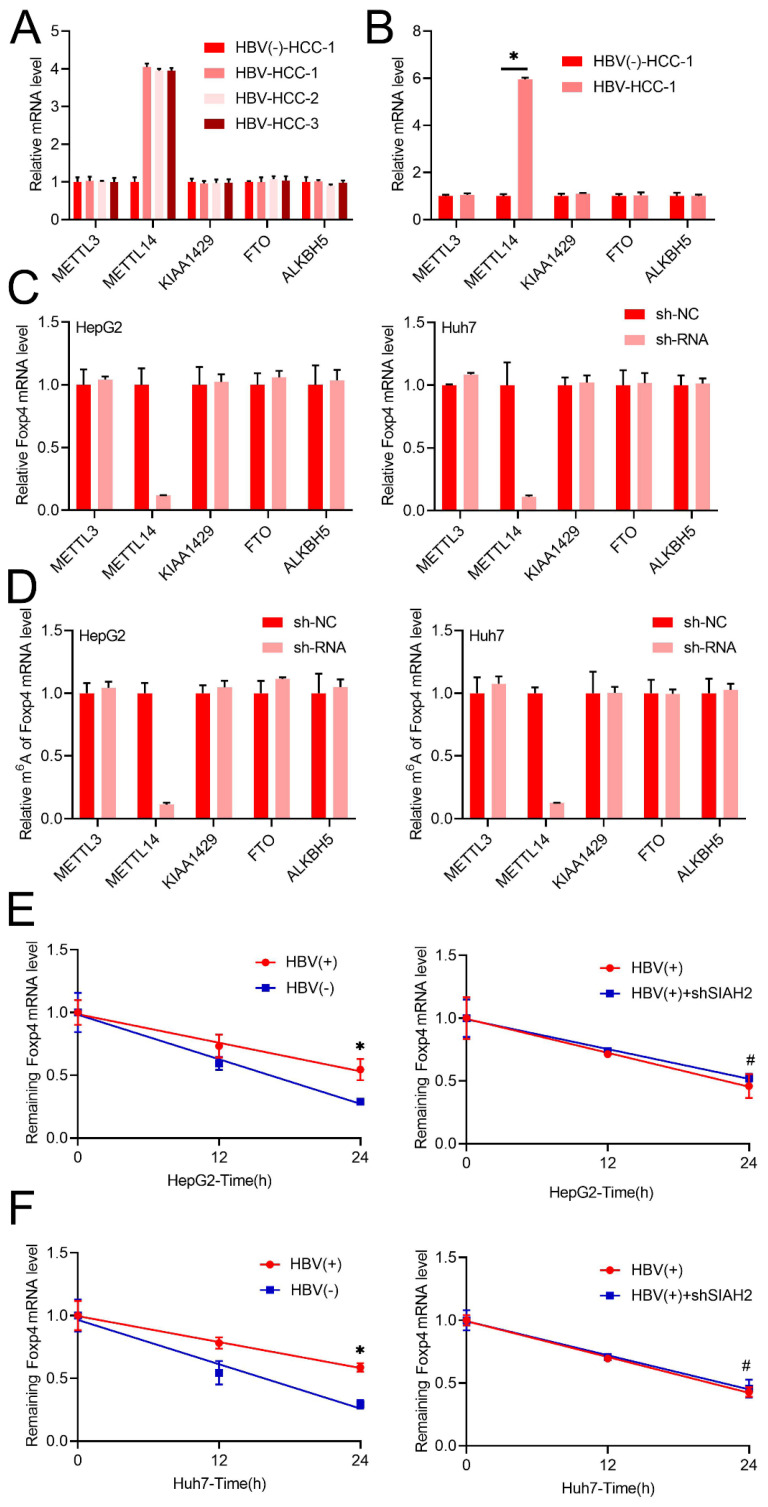
** HBV enhances FOXP4 mRNA stability via modulation of m6A modification.** (A and B) The mRNA levels of key enzymes involved in m6A modification were measured in the liver biopsies and PHH infected with HBV. (C) qRT-PCR quantification of Foxp4 mRNA levels in HCC cells with shMETTL3/METTL14/KIAA1429/FTO/ALKBH5. (D). MeRIP-qRT-PCR analysis was m^6^A level of Foxp4 mRNA in HCC cells with shMETTL3/METTL14/KIAA1429/FTO/ALKBH5. (E) qRT-PCR analysis of remaining Foxp4 mRNA. (F) The HBV transfected HCC cells were depleted for remaining Foxp4 mRNA. *P < 0.05.

Following we noted that METTL14 mRNA levels increased significantly in both chronic HBV patient samples and primary human hepatocytes infected with HBV, other m6A regulators such as METTL3, KIAA1429, ALKBH5, and FTO remained unchanged (Figure 2A and B). Interestingly, only knock down METTL14, while not other m6A regulators such as METTL3, KIAA1429, ALKBH5, and FTO decreased m6A and mRNA level of Foxp4 in HCC cells (Figure 2C and D). Additionally, transient HBV expression was shown to increase the half-life of Foxp4 mRNA, while the absence of METTL14 prevented HBV-induced changes in Foxp4 mRNA levels (Figure 2E and F).

### FoxP4 potentiates the malignant phenotype of HCC cells

**Figure 3:**
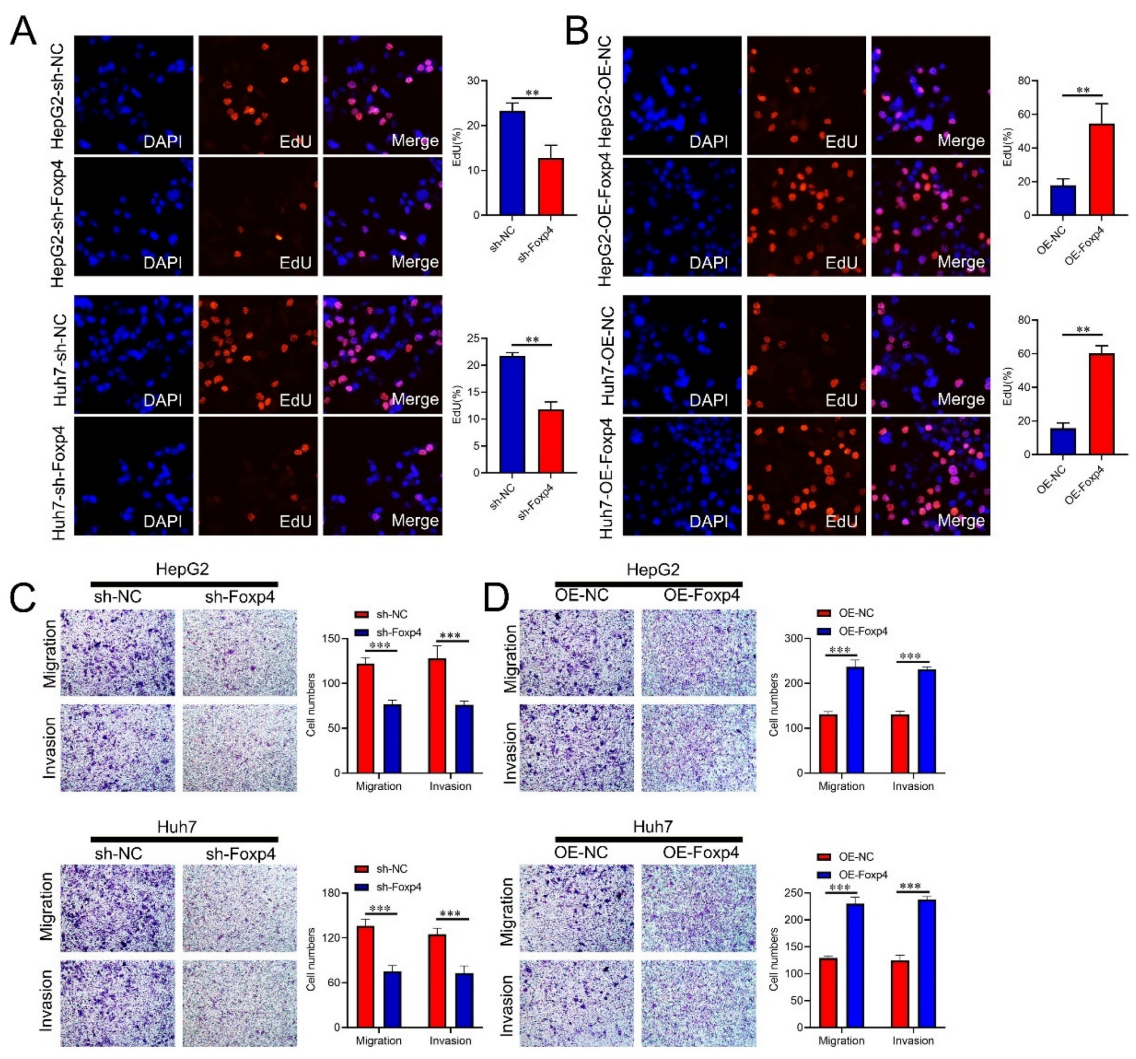
** FoxP4 potentiates the malignant phenotype of HCC cells. (A-B)** Cell viability of HCC cells was examined by EdU assays, magnification x 400. **(C and D)** The transwell assays were performed in indicated HCC cells. Magnification x 400. ** P < 0.01; *** P < 0.001.

The Foxp4 knockdown significantly reduced the proportion of EdU-positive cells in HCC cells compared to the control group (Figure 3A), while Foxp4 overexpression had the opposite effect (Figure 3B). Transwell assays demonstrated that downregulation of Foxp4 significantly inhibited cell migration and invasion (Figure 3C), while overexpression of Foxp4 enhanced these abilities (Figure 3D).

### HBV stimulates the PI3K/AKT pathway by enhancing m6A modification of Foxp4 mRNA

**Figure 4:**
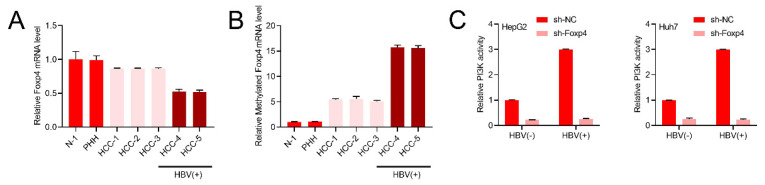
** HBV stimulates the PI3K/AKT pathway by enhancing m6A modification of Foxp4 mRNA.** (A) qRT-PCR quantification of Foxp4 mRNA levels. (B) MeRIP-qRT-PCR analysis of samples in (A). (C) PI3K activity in Indicated HCC cells.

Our research revealed a significant decrease in Foxp4 mRNA expression levels in both HBV-positive and negative HCC patients (Figure 4A). m6A methylation of Foxp4 mRNA was found to be elevated in all HCC samples, with HBV-positive patients exhibiting higher levels compared to HBV-negative cases (Figure 4B). Further investigation into the PI3K activity in HBV expressing cells supported this notion (Figure 4C).

## Discussion

Hepatitis B virus has been identified as playing a crucial role in promoting tumor development by affecting the epigenetic regulation of host cells. However, the extent to which newly defined epitranscriptomics regulation is involved in HBV-induced epigenetic instability remains largely unexplored 21-25. The inhibition of Foxp4 by HBV may serve as one of the mechanisms that accelerates tumor development in cases of chronic virus infection. It has been observed that HBV transfection leads to alterations in cellular m6A profiling, prompting further investigation into how HBV specifically impacts m6A modification of cellular RNAs. Interestingly, research has found that HBV infection results in an increase in METTL14 expression levels in chronic HBV patients, HBV-positive hepatocellular carcinoma (HCC) patients, and primary human hepatocytes infected with HBV. This raises the compelling possibility that the upregulation of METTL14 expression by HBV is linked to the development of liver tumors. This hypothesis was supported by analyses showing changes in METTL14 expression levels in HBV-negative HCC patient samples and HepG2 cells transfected with HBV. Furthermore, studies demonstrate that m6A modification plays a crucial role in regulating mRNA stability, splicing, nuclear export, and translation of target mRNAs. Specifically, the increase in Foxp4 mRNA levels induced by METTL14 was found to be primarily due to the slowed degradation of Foxp4 mRNA, orchestrated by the m6A reader protein YTHDF1 26, resulting in elevated Foxp4 protein levels. These findings strongly suggest that HBV-induced upregulation of METTL14 in normal liver cells promotes m6A modification of Foxp4 mRNA, leading to increased RNA stability and potentially contributing to the development of HCC.

Interferon-alpha is a powerful tool in the treatment of HBV infection 27-31. Adjuvant interferon therapy following hepatectomy has been proven to reduce the recurrence of HBV-related HCC and enhance overall survival rates in patients 30,31. Our research has discovered increased expression of Foxp4 during HBV infection, whether it potentially inhibits p-IRF-3 nuclear import and disrupts the IFN signaling pathway has not been studied and is worth exploring in the future.

The fork head box (FOX) protein, specifically FOXP4, is a crucial player in various biological processes, particularly in HCC 14-17. This research sheds light on the critical role of FOXP4 in the HBV-HCC progression, providing valuable insights for potential clinic-therapy interventions. This study marks a significant contribution to understanding the complex functions of FOXP4 in cancer development.

Shortcomings of the paper, we are unable to obtain sufficient clinical samples for validation the correlation of expression profiles of METTL14, N6-Methyladenosine, and FOXP4 with any of clinic parameters. In summary, our findings shed light on how HBV manipulates host gene expression to facilitate long-term infection by influencing m6A modification processes. Specifically, the virus-induced modification of Foxp4 through m6A regulation may play a role in the development of liver cancer linked to HBV infection. These insights reveal new pathways for potential therapeutic interventions against HBV-related hepatocarcinogenesis.

## Supplementary Material

Supplementary figure and table.

## Figures and Tables

**Figure 5:**
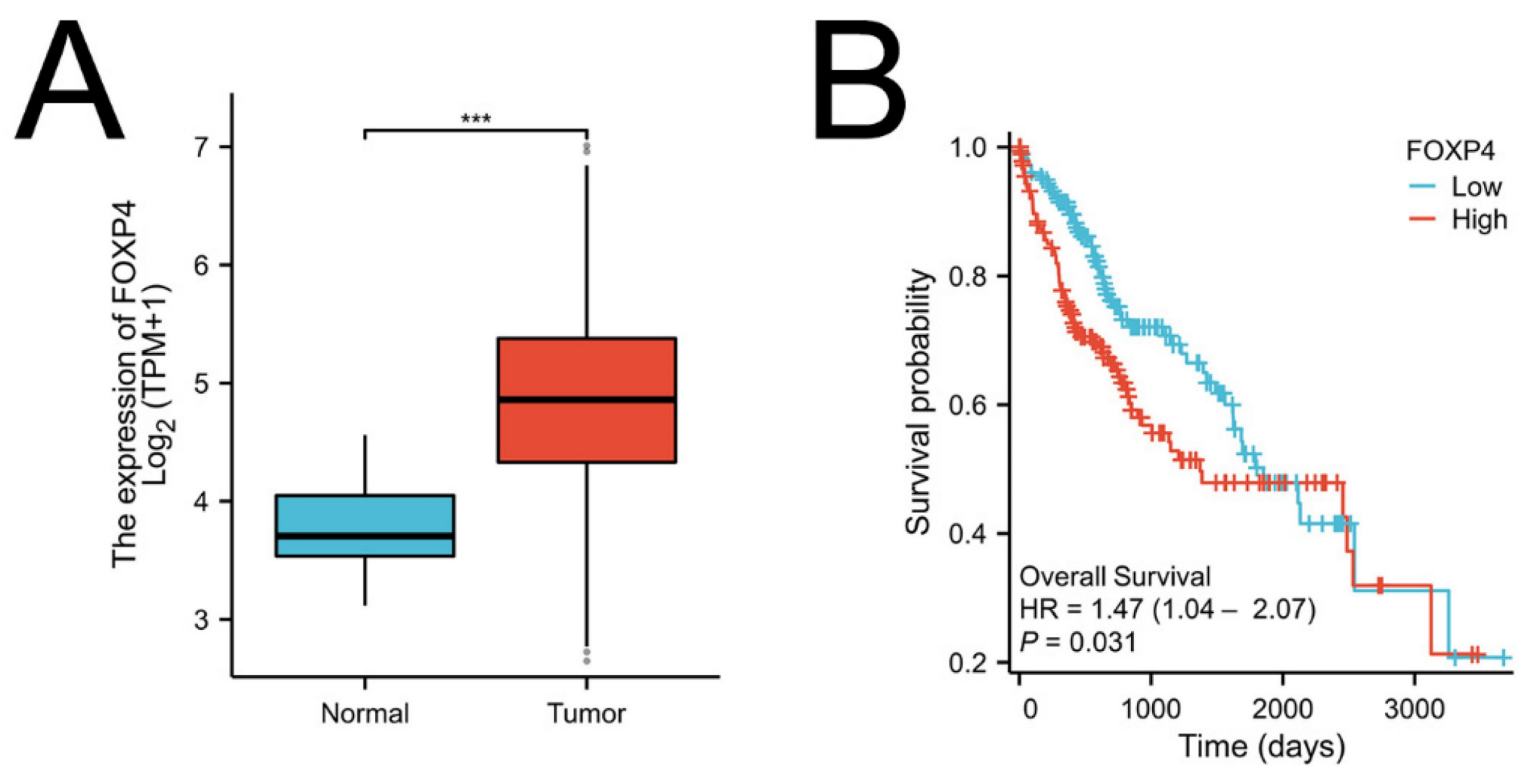
** Foxp4 levels linked to poor survival in HCC.** (A) Foxp4 mRNA expression in HCC tissues and normal tissues. (B) Kaplan-Meier curve of Foxp4 mRNA expression for overall survival (OS) in HCC. The high/low Foxp4 group was cut based on the mean expression in HCC.The high Foxp4 group=185, the low Foxp4 group=184. *** P < 0.001.
