# Supplementary material for: METTL14 Induced N6-Methyladenosine Modification of FOXP4 mRNA in HBV-HCC
Source: J Cancer. 2024 Oct 14;15(19):6232–8. doi: 10.7150/jca.101385 (PMC11540497; doi:10.7150/jca.101385)
Supplement: Supplementary file 1 — Supplementary figure and table. [file jcav15p6232s1.pdf]

Supplement information

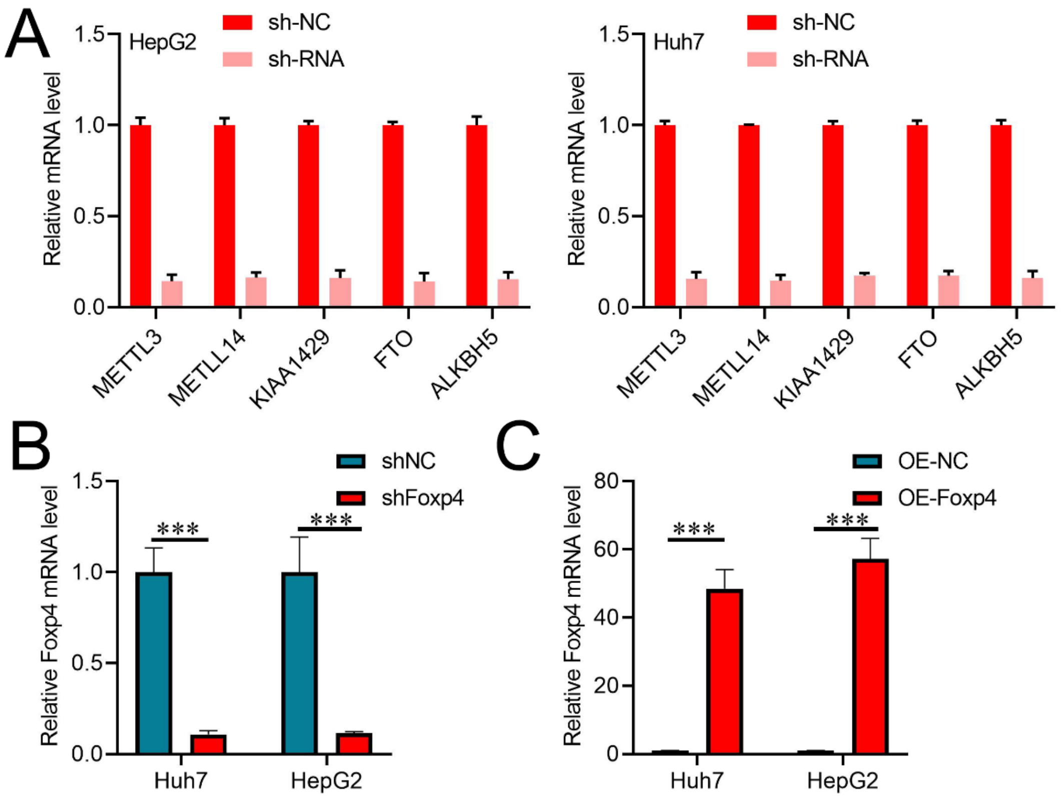

Supplement Figure 1

(A) qRT-PCR quantification of METTL3,METTL14,KIAA1429,FTO,and ALKBH5 mRNA levels in HCC cells with sh-METTL3,METTL14,KIAA1429,FTO,and ALKBH5. (B and C) qRT-PCR quantification of Foxp4 mRNA levels in HCC cells with sh-Foxp4 or OE-Foxp4. Mean ± SD of three independent experiments. *P<0.05; ** P < 0.01; *** P < 0.001

Table S1: shRNA sequences used for genes knockdown

ShRNA	Sense	Antisense
METTL3	GGACCAAGGAAGAGUGCAUdTdT	AUGCACUCUUCCUUGGUCCdTdT
METTL14	GGGAGAGUAUGCUUGCGAATT	UUCGCAAGCAUACUCUCCCTT
KIAA1429	GGAUCAGACUGUGAGGGUUTT	AACCCUCACAGUCUGAUCCTT
FTO	GGGAGAACAUUCUUGUCAUTT	AUGACAAGAAUGUUCUCCCTT
ALKBH5	AGCAGAUAAAGAUGUUCAAdTdT	UUGAACAUCUUUAUCUGCUdTdT
FOXP4	GGAAAGAAGGAGUAUCUAATdT	UUAGAUACUCCUUCUUUCCTdT
